# Dynamical behavior of neural ensembles: bifurcation analysis approach

**DOI:** 10.1186/1471-2202-12-S1-P6

**Published:** 2011-07-18

**Authors:** Ramin Azodi, Mohamad Ghadami

**Affiliations:** 1 Department of Mechatronics, University of Tehran-Kish International Campus, Kish Island, 79416-55665, Iran

## 

Computational analysis of neural networks has a significant effect to recognise the behaviour of synaptic neurons. In the past few years, dynamical systems and nonlinear phenomena related to dynamics of neurons achieve much attention. These structures are typically modeled by set of ordinary differential equations (ODE) which represent a well understanding approach in order to explore dynamic behavior of the network [1]. In fact, these equations are mathematically consistent with the performance of biological structures. One of the parameters which possesses prominent effect on the whole activity of the ensembles is the strength among neural networks that appears in ODE equations. Changing in strength of a typical network might lead to transition between phases which are identified by different local and global bifurcations. In addition, the main aspect which leads to complexity of these structures is interactions between intrinsically oscillatory neurons and their influence on each other [2]. These interactions could be considered as coupled systems among neurons or network oscillators. In coupled systems each oscillator is determined by a coupling strength and the states of oscillatory networks. Therefore, bifurcation analysis can be useful method to be taken into account.

In this work, we applied bifurcation theory as a promising mathematical method to reveal the dynamical behavior of a biological system (neural network zoom for this purpose). For this purpose the computational of local and global bifurcations has been done using AUTO software’s package [3]. Knowing that Saddle Node bifurcation (SNB) is used to detect stability of the networks, whereas creations of periodic solutions are corresponds to Hopf bifurcation (HB). Also, it should be noted that heteroclinic bifurcation is utilized to investigate sequential switching activity in the neural ensembles in phase space [4]. All of these approaches employed in analyzing our models. Moreover, the synchronous activity of neural populations could be extracted from ODE equations.

**Figure 1:**
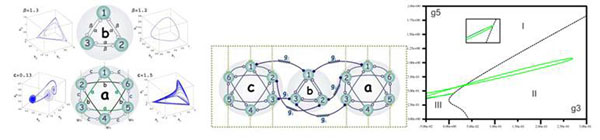
**A.** Phase space diagram of each ensembles. **B.** Schematic diagram of ensemble. **C.** The bifurcation diagram of two typical electrical coupling.

Through the simulation of a single ensemble, we concluded that, increase in number of neurons and consequently connections between them in ensembles leads to more complexity in dynamical behavior of the system (Figure 1A). We also found that if we consider some specific neurons with dominant coupling strength in comparison with oscillatory neurons in the whole systems, the ensembles will experience instability in some cases. Furthermore, in this research electrical coupling between ensembles had considerable importance for us. Drawing bifurcation diagram for different kinds of ensembles let us to deduced that, when the values of electrical couplings becomes near to each other (Figures 1B and 1C), It shows the chaotic behavior of the system. We also got some interesting result about the effect of strength intensity on intrinsically oscillatory neurons. Further researches on this subject are in progress.
